# Integrating traditional omics and machine learning approaches to identify microbial biomarkers and therapeutic targets in pediatric inflammatory bowel disease

**DOI:** 10.3389/fphar.2025.1545392

**Published:** 2025-04-14

**Authors:** Lanlan Li, XuZai Deng, Shuge Wang, Tao Huang

**Affiliations:** ^1^ Department of Pediatrics, Tianyou Hospital Affiliated to Wuhan University of Science and Technology, Wuhan, China; ^2^ Department of Pediatrics, Maternal and Child Health Hospital of Hubei Province, Tongji Medical College, Huazhong University of Science and Technology, Wuhan, China

**Keywords:** pediatric IBD, biomarkers, RNA-Seq, machine learning, therapeutic targets, reproducibility

## Abstract

**Background:**

Pediatric inflammatory bowel disease (IBD), especially Crohn’s disease, significantly affects gut health and quality of life. Although gut microbiome research has advanced, identifying reliable biomarkers remains difficult due to microbial complexity.

**Methods:**

We used RNA-seq-based microbial profiling and machine learning (ML) to find robust biomarkers in pediatric IBD. Microbial taxa were profiled at phylum, genus, and species levels using kraken2 on Crohn’s disease and non-IBD ileal biopsies. We performed abundance-based analyses and applied four ML models (Logistic Regression, Random Forest, Support Vector Machine, XGBoost) to detect discriminative taxa. An independent cohort of 36 pediatric stool samples assessed by 16S rRNA sequencing validated top ML results.

**Results:**

Traditional abundance-based methods showed compositional shifts but identified few consistently significant taxa. ML models had better discriminatory performance, with XGBoost outperforming others and pinpointing Orthotospovirus and Vescimonas as key genera. These findings were confirmed in the validation cohort, where only one traditionally noted genus, *Actinomyces*, maintained significance.

**Discussion:**

Integrating conventional omics with AI-driven analytics boosts reproducibility and clinical relevance of microbial biomarker discovery, opening new possibilities for targeted therapies and precision medicine in pediatric IBD.

## Introduction

Pediatric inflammatory bowel disease (IBD), encompassing Crohn’s disease (CD) and ulcerative colitis (UC), remains a formidable clinical and research challenge due to its complex etiology, variable presentation, and substantial impact on growth and development. Unlike adult-onset IBD, pediatric IBD can have more extensive intestinal involvement and a higher disease burden, often manifesting as delayed puberty, impaired growth, and reduced quality of life. Early and accurate diagnosis, alongside effective therapeutic interventions, is crucial for mitigating long-term complications and improving patient outcomes. However, the current diagnostic paradigm—primarily reliant on invasive endoscopic examinations and histopathological assessments—offers limited non-invasive biomarkers capable of reliably differentiating pediatric IBD from other gastrointestinal conditions.

Advancements in omics technologies, particularly RNA-sequencing (RNA-seq), have substantially enhanced our understanding of the gut microbiome’s role in health and disease (Beaudry et al., 2016; Lloyd-Price et al., 2016). By capturing the functional and taxonomic composition of microbial communities, RNA-seq has facilitated the identification of candidate biomarkers and putative drug targets (Franzosa et al., 2014). Yet, while such abundance-based analyses have highlighted certain taxa, reproducibility and consistency across independent cohorts remain vexing issues. Many proposed microbial biomarkers fail to display stable, cross-study validation, hampering their clinical applicability and limiting insights into potential therapeutic mechanisms (Haberman et al., 2014; Shapiro et al., 2018).

Although advancements in omics-based technologies have yielded valuable insights into the gut microbiome’s contribution to IBD, traditional abundance-based approaches often fail to identify reproducible biomarkers, resulting in a “reproducibility crisis.” This reproducibility crisis underscores the need for integrative strategies that extend beyond conventional abundance-based approaches. Artificial intelligence (AI) and machine learning (ML) offer a powerful complement to traditional methods, capable of discerning subtle patterns and complex interactions within large, multi-dimensional datasets (Topol, 2019; Beam and Kohane, 2018). ML models, such as Random Forest, Support Vector Machines (SVM), and gradient-boosting algorithms like XGBoost, can sift through vast numbers of features—including microbial taxa at various taxonomic levels—and prioritize the most informative markers with high predictive value. Applying similar strategies to pediatric IBD could enhance early diagnosis, uncover novel therapeutic targets, and inform personalized interventions (Esteva et al., 2019).

The gut microbiome’s complexity in pediatric IBD is further complicated by age-related factors. Children’s microbiotas are dynamic, influenced by diet, early-life exposures, and ongoing maturation of the immune system (Yatsunenko et al., 2012). Such complexity demands robust analytical tools that can integrate biological knowledge with computational efficiency. Traditional omics approaches offer depth and mechanistic understanding, while ML provides scalability, pattern recognition, and improved predictive performance when confronted with heterogeneous and noisy data (Franzosa et al., 2019; Laukens et al., 2016).

Moreover, identifying biomarkers that translate into actionable drug targets requires a comprehensive approach that moves beyond static abundance measures. RNA-seq data can reveal microbial gene expression patterns, shedding light on metabolic pathways and potential therapeutic mechanisms (Paramsothy et al., 2017). By focusing on microbial taxa consistently linked to pediatric IBD, researchers may pinpoint targets for microbiome-modulating therapies—such as probiotics, prebiotics, fecal microbiota transplantation (FMT), or even metabolite-targeted interventions—that hold promise in complementing or enhancing existing pharmacological treatments (Sainath et al., 2020; Sinha et al., 2017).

Building on previous efforts to integrate computational and experimental methods in microbiome research, this study demonstrates the potential of combining RNA-seq-based microbial profiling with machine learning (ML) to enhance biomarker reproducibility and identify promising therapeutic targets in pediatric IBD. In this study, we combined traditional RNA-seq microbial profiling with ML-driven approaches to systematically identify reliable microbial signatures of pediatric IBD. By contrasting abundance-based statistical methods with multiple ML classifiers—including Logistic Regression, Random Forest, Support Vector Machine (SVM), and XGBoost—we aimed to address the synergistic value of integrating complementary methodologies. Ultimately, this approach seeks to enhance reproducibility in biomarker discovery and inform precision medicine strategies for pediatric IBD.

## Materials and methods

### Study cohorts and sample collection

We analyzed ileal biopsy RNA-seq data obtained from pediatric patients diagnosed with Crohn’s disease (CD) and age-matched non-IBD controls. The primary dataset comprised 245 pediatric ileal biopsy samples originally described in publicly available repositories (GSE93624) (Schloss et al., 2011). In addition to this primary dataset, an independent validation cohort was assembled comprising 36 pediatric stool samples (19 IBD, 17 non-IBD), collected prospectively and processed using 16S rRNA gene sequencing to test the reproducibility of identified microbial biomarkers (Caporaso et al., 2012). Our protocols to include: (i) standardized fasting requirements (at least 8 h prior to biopsy) when clinically permissible, (ii) avoidance or documentation of probiotics and specific medications within 4 weeks prior to sampling, and (iii) adherence to uniform dietary guidelines where possible. Non-IBD controls included children undergoing ileal biopsy for reasons unrelated to IBD who exhibited normal histopathology and no signs of inflammatory disorders (Supplementary Table S1). Samples were immediately flash-frozen and stored at −80°C to preserve microbial integrity. Stool collection also followed standardized protocols, including instructions for home collection kits, immediate cooling, and rapid transfer to the lab, where samples were stored at −80°C before 16S rRNA gene sequencing. Patients were enrolled following informed consent and in accordance with the ethical guidelines and approval of the Tianyou Hospital Affiliated to Wuhan University of Science and Technology Review Board.

### RNA-seq data processing and microbial profiling

Raw paired-end RNA-seq reads were quality-checked using FastQC and filtered to remove low-quality reads and adapter contamination with Trimmomatic (Bolger et al., 2014), applying default parameters for Illumina data. Host reads were removed by mapping against the human reference genome (GRCh38) using Hisat2 (Kim et al., 2015), retaining only non-host reads for downstream microbiome profiling. Microbial sequences were taxonomically classified at phylum, genus, and species levels using kraken2 (Wood and Salzberg, 2014) with a comprehensive reference database, ensuring robust identification of bacterial, viral, and fungal taxa. To generate a normalized microbial abundance table, we calculated the relative abundance of each taxon by dividing raw counts by the total number of microbial reads per sample. Only taxa present in at least 10% of samples were retained to minimize the influence of rare and potentially spurious features.

### Alpha and beta diversity analyses

To assess within-sample microbial diversity (alpha diversity), we computed the Shannon index using vegan R package functions (Oksanen 2020). Between-sample compositional differences (beta diversity) were evaluated using the Bray-Curtis dissimilarity metric, followed by Principal Coordinates Analysis (PCoA) for visualization. Group comparisons were performed using PERMANOVA (adonis function in vegan) to test for significant shifts in community structure (Anderson, 2017).

### Traditional abundance-based comparisons

To identify differentially abundant taxa between pediatric CD and non-IBD controls using conventional approaches, we employed non-parametric tests (Wilcoxon rank-sum) on relative abundance data, adjusting for multiple comparisons with the Benjamini-Hochberg method (Benjamini and Hochberg, 1995). Taxa displaying a false discovery rate (FDR)-adjusted p-value <0.05 were considered statistically significant. Selected taxa were visualized with boxplots and stacked barplots to depict compositional differences at phylum and genus levels (Wickham, 2016).

### Machine learning approaches for biomarker discovery

To enhance biomarker discovery, we applied four ML algorithms: Logistic Regression, Random Forest, SVM, and XGBoost. We performed hyperparameter tuning via grid search and 5-fold cross-validation for each model. For example, our SVM tested multiple kernel types (linear, polynomial, radial basis), while our XGBoost pipeline varied learning rates (0.01–0.3), max_depth (Franzosa et al., 2014; Haberman et al., 2014; Shapiro et al., 2018; Topol, 2019; Beam and Kohane, 2018; Esteva et al., 2019; Yatsunenko et al., 2012; Franzosa et al., 2019), and regularization parameters (lambda, alpha). Model performance was evaluated on a 70/30 train/test split, with Area Under the Receiver Operating Characteristic Curve (AUC), accuracy, sensitivity, and specificity as key metrics (Chen and Guestrin, 2016). Feature selection was conducted using a forward selection strategy, gradually adding taxa that improved classification performance until no further gain was observed. Models were evaluated on the testing set, and their performance was compared based on AUC, accuracy, sensitivity, and specificity (Kuhn, 2008). The best-performing model was then applied to identify the most informative taxa for distinguishing pediatric CD from non-IBD controls (Supplementary Table S2).

### Independent validation and reproducibility testing

To validate the identified biomarkers, we analyzed the independent pediatric cohort of 36 stool samples processed via 16S rRNA amplicon sequencing. This cohort underwent similar quality control and taxonomic assignment steps using QIIME2 and DADA2 (Callahan et al., 2016), ensuring consistent methods for feature representation. The taxa highlighted by ML-based approaches were examined in this validation cohort to assess reproducibility. Taxa derived from traditional abundance-based methods were also tested, facilitating a direct comparison of stability and clinical relevance (Segata et al., 2011).

### Statistical analyses and software

All statistical analyses were performed in R (version 4.2.2). Multiple packages, including phyloseq for microbiome data handling (McMurdie and Holmes, 2013), vegan for diversity analyses (Oksanen 2020), and caret for machine learning workflows (Kuhn and Johnson, 2013), were employed. Visualization was completed using python3 for differential abundance results. Unless otherwise stated, p-values were two-tailed, and p < 0.05 was considered significant.

## Results

### Microbial community composition at phylum and genus levels

**FIGURE 1:**
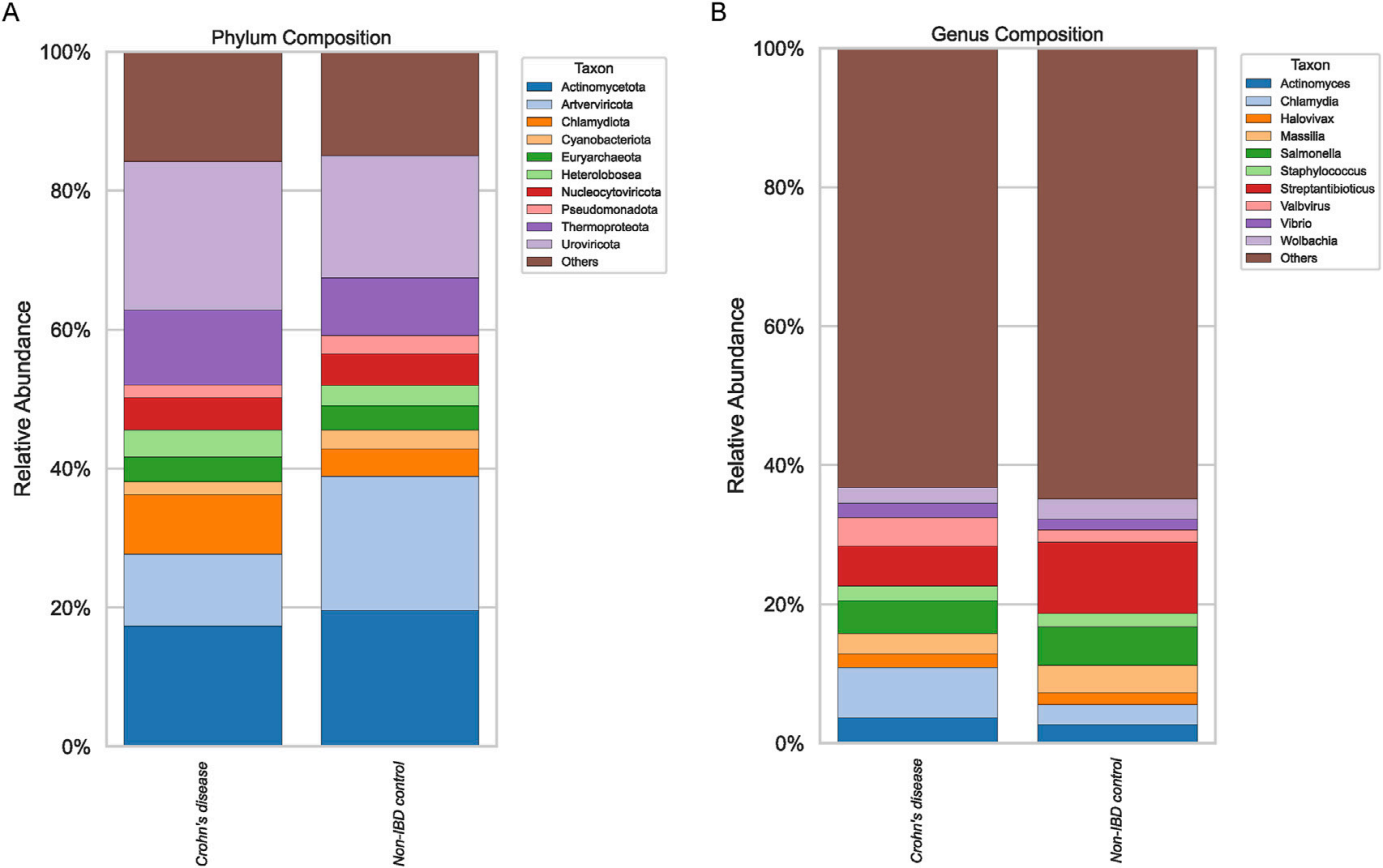
Phylum and Genus Composition. Stacked barplots comparing the relative abundance at phylum level **(A)** and genus level **(B)** between Crohn’s disease and non-IBD control groups.

Compositional profiling of pediatric Crohn’s disease and non-IBD ileal biopsy samples revealed distinct microbiome signatures at both the phylum and genus levels (Figure 1). While the overall phylum distribution appeared dominated by a few core taxa in both groups, subtle shifts were evident, suggesting alterations in microbial community structures associated with disease status. At the genus level, stacked barplots indicated that certain taxa were enriched in either pediatric IBD or non-IBD controls, yet the magnitude and consistency of these differences varied across samples.

### Alpha and beta diversity analyses at the genus level

**FIGURE 2:**
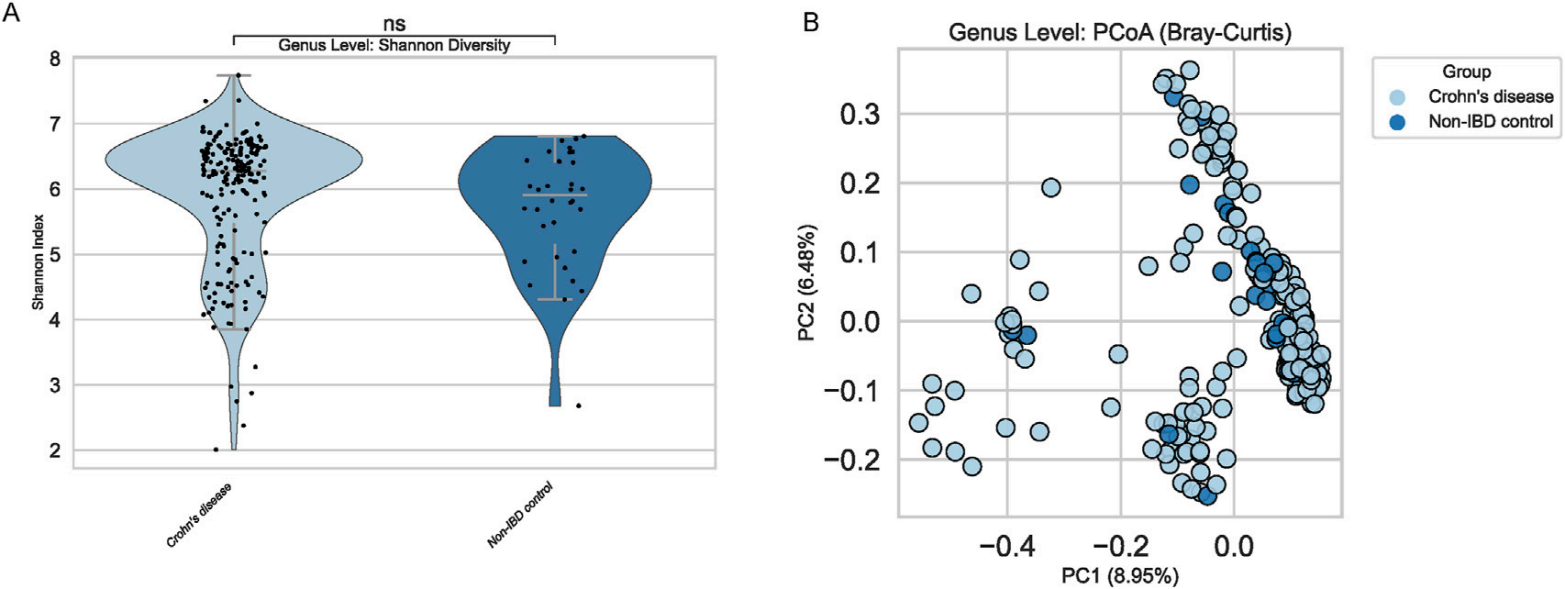
Alpha and Beta Diversity at Genus Level. **(A)** Violin plot of Shannon index shows no significant differences between groups. **(B)** PCoA plot (Bray-Curtis) indicates no clear clustering by disease status.

Alpha diversity metrics, such as the Shannon index, showed no significant differences between pediatric IBD and non-IBD groups (Figure 2A). Both cohorts exhibited comparable within-sample microbial complexity, implying that reduced richness or evenness may not be a defining feature of pediatric IBD in this dataset. Conversely, beta diversity analyses using Bray-Curtis distances suggested mild compositional shifts (Figure 2B). Principal Coordinates Analysis (PCoA) showed a trend of clustering by disease status, albeit with notable overlap, highlighting the subtlety of the microbial distinctions.

### Identifying taxa with traditional abundance-based methods

**FIGURE 3:**
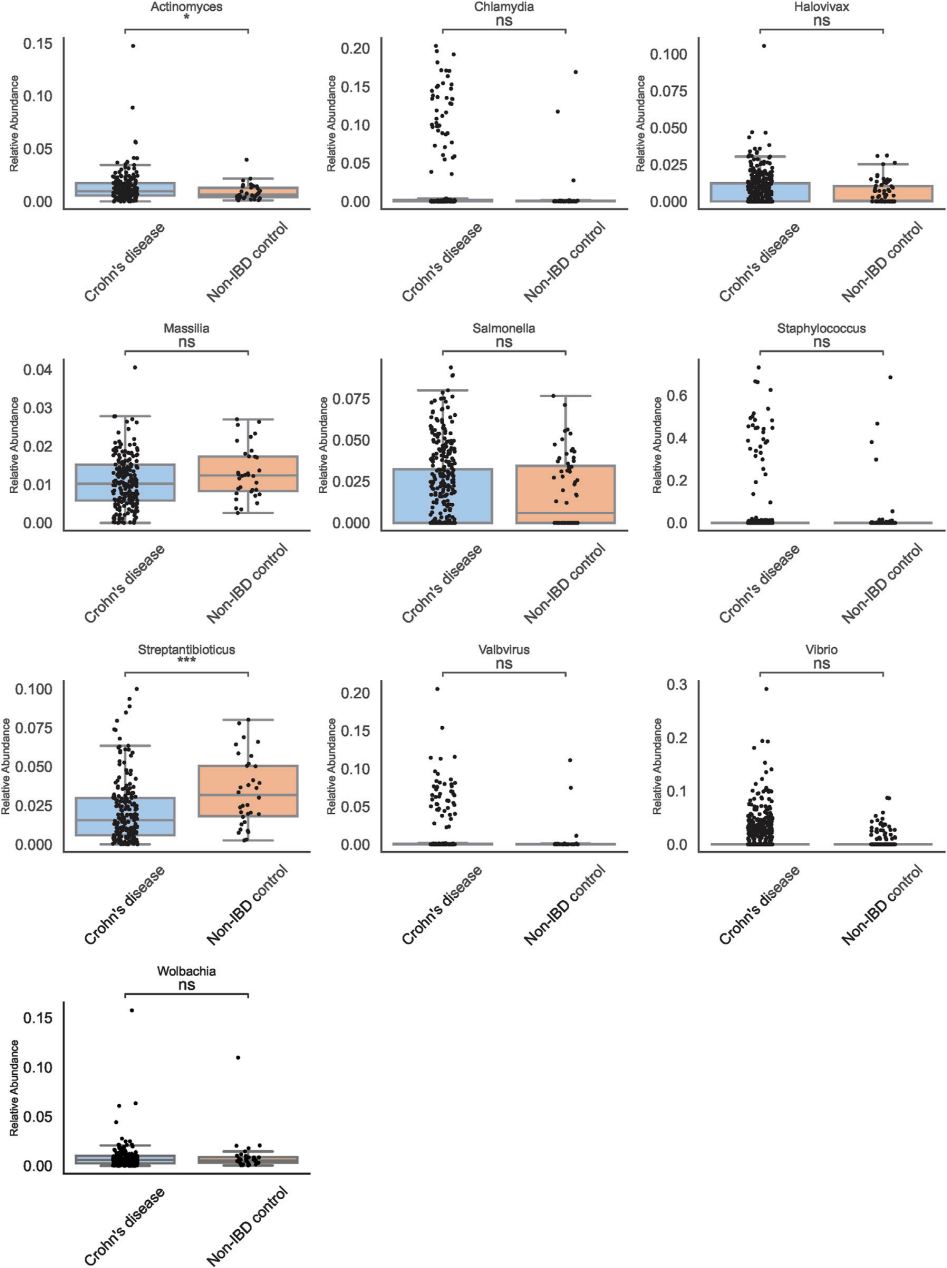
Genus-Level Boxplots from Traditional Abundance Analyses. Boxplots for selected genera reveal *Actinomyces* and Streptantibioticus as differentially abundant, but only *Actinomyces* is significant.

Traditional abundance-based comparisons identified a shortlist of genera that appeared differentially abundant between pediatric IBD and non-IBD samples. Among these, *Actinomyces* and Streptantibioticus emerged as candidates of interest (Figure 3). Boxplots of these genera revealed that *Actinomyces* displayed a more consistent pattern of enrichment in pediatric IBD, whereas Streptantibioticus showed differences but lacked robust statistical significance or consistency. These findings underscored the challenges inherent in relying solely on abundance-based methods, as initial signals may not always translate into stable biomarkers.

### Machine learning-based biomarker discovery

**FIGURE 4:**
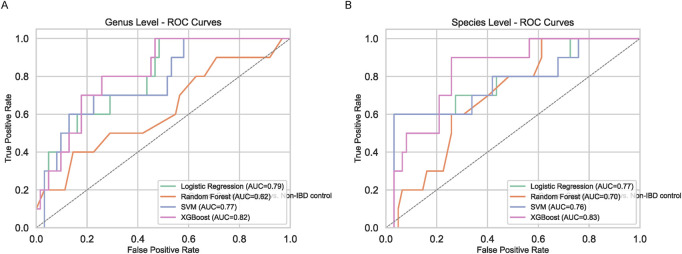
Machine Learning-Based Classification. ROC curves for Logistic Regression, Random Forest, SVM, and XGBoost at genus **(A)** and species **(B)** levels. XGBoost outperforms others.

**FIGURE 5:**
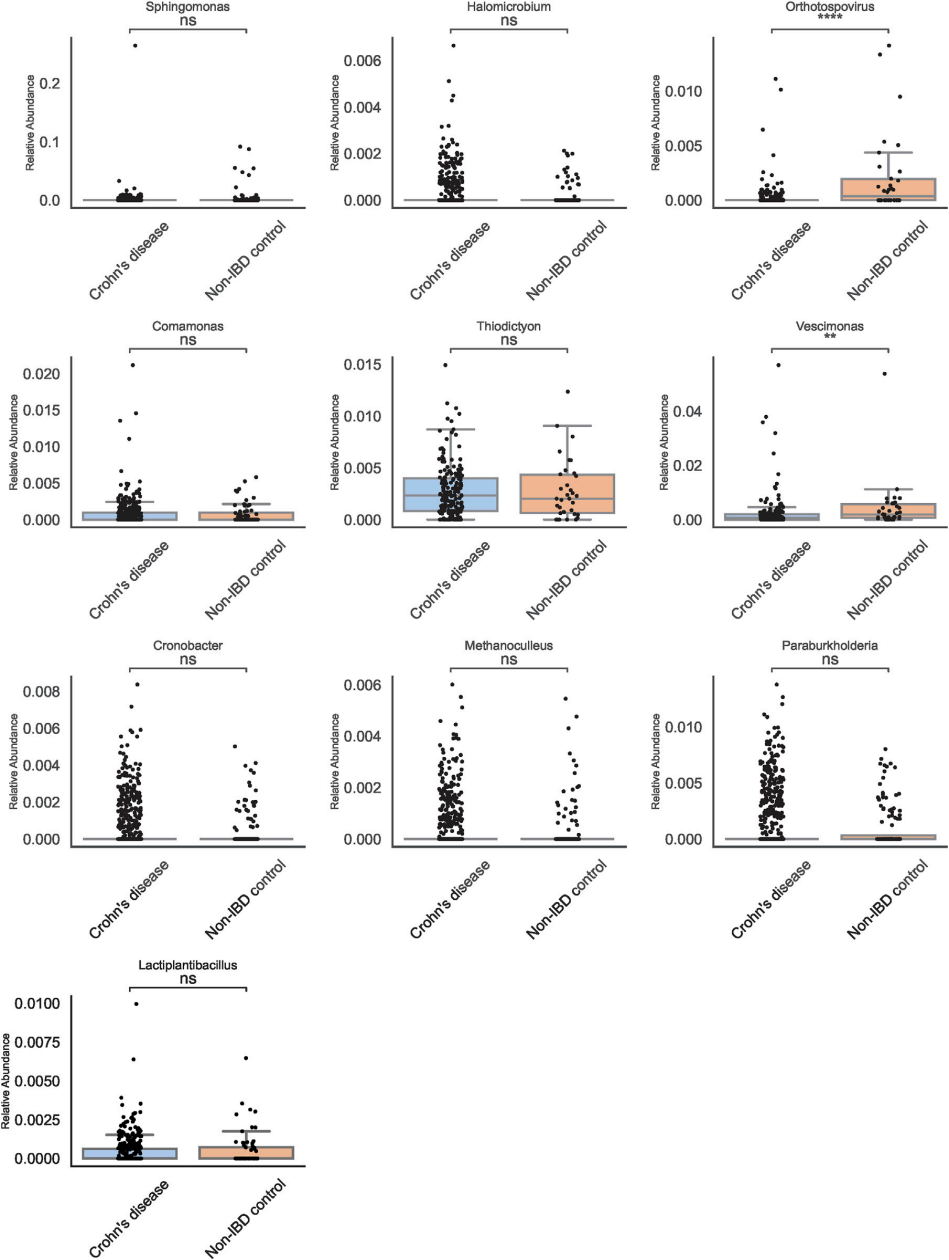
Key Genera Identified by XGBoost. Boxplots show Orthotospovirus (****) and Vescimonas (**) as top discriminators. These taxa exhibit robust differences between Crohn’s disease and controls.

To enhance biomarker reproducibility, multiple machine learning (ML) classifiers were employed at both genus and species levels (Figure 4). While Logistic Regression, Random Forest, and SVM provided modest improvements over traditional methods, XGBoost outperformed all other models, demonstrating higher accuracy and a more pronounced ability to discriminate pediatric IBD from non-IBD controls. Feature importance analyses identified Orthotospovirus and Vescimonas at the genus level as key discriminators (Figure 5). Unlike many taxa highlighted by conventional abundance approaches, these XGBoost-selected genera exhibited robust and reproducible differences, suggesting they may serve as reliable biomarkers.

### Independent validation in a separate pediatric cohort

**FIGURE 6:**
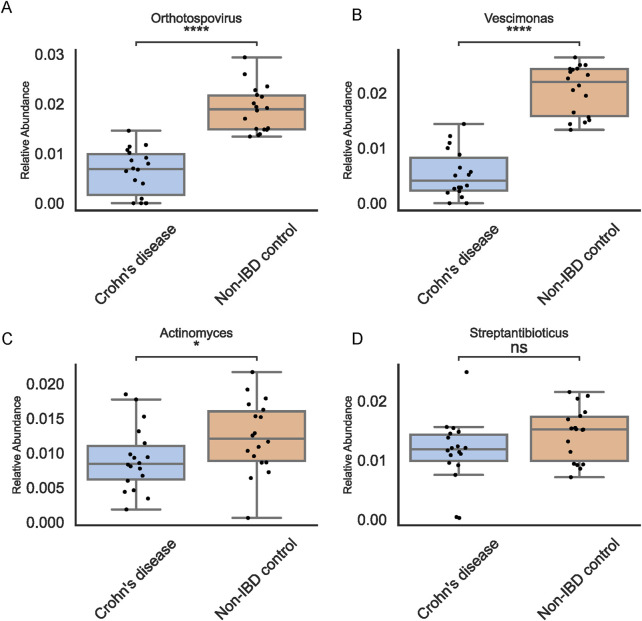
Independent Validation in 36 Pediatric Fecal Samples. Boxplots for top genera in the validation set confirm consistent trends for Orthotospovirus **(A)**, Vescimonas **(B)**, *Actinomyces* **(C)**, and Streptantibioticus **(D)** highlighting reproducibility of ML-based findings and indicating potential therapeutic targets.

To test the reproducibility and clinical relevance of the identified biomarkers, we examined an independent cohort of 36 pediatric stool samples using 16S rRNA sequencing (Figure 6). Orthotospovirus and Vescimonas maintained consistent trends in this external dataset, supporting their status as stable biomarkers. Notably, among the taxa initially suggested by abundance-based methods, only *Actinomyces* retained its observed pattern. This independent validation emphasized the value of combining traditional and ML-driven approaches to achieve reproducible and clinically meaningful results.

## Discussion

This study demonstrates how integrating traditional omics analyses with AI-driven machine learning can refine the search for robust microbial biomarkers and potential therapeutic targets in pediatric IBD. While traditional abundance-based comparisons provided an initial shortlist of candidate taxa, their reproducibility proved limited. *Actinomyces* stood out as the sole traditional candidate maintaining consistent patterns across datasets, underscoring the rarity of stable biomarkers emerging from conventional methods alone. Our findings underscore XGBoost’s advantages for this dataset, including its capacity to handle imbalanced classes, accommodate complex feature interactions, and integrate regularization to prevent overfitting. These attributes appear especially beneficial in microbiome studies where underlying microbial signals may be subtle or masked by high inter-individual variability. Identifying Orthotospovirus and Vescimonas as key discriminators speaks to the capacity of ML tools to capture complex interactions that might be overlooked by simpler statistical approaches. By systematically incorporating feature selection, cross-validation, and performance metrics, the ML framework not only improved classification accuracy but also enhanced the likelihood that identified taxa reflect underlying disease mechanisms rather than spurious associations.

The independent validation further corroborated the strength of ML-driven discoveries. While one genus from the traditional approach retained its trend, the consistent appearance of Orthotospovirus and Vescimonas across cohorts underscores their potential involvement in pediatric IBD pathogenesis. Orthotospovirus, traditionally known as a plant-infecting viral genus, may have unrecognized roles in human health through complex interactions within the gut ecosystem, while Vescimonas, a relatively understudied bacterial genus, could influence immune regulation or mucosal barrier integrity (De Oliveira et al., 2018; Huang et al., 2024). Such biomarkers can serve as entry points for investigating how shifts in the microbial community affect mucosal inflammation and immune modulation, ultimately guiding microbiome-targeted therapies—whether through dietary modifications, microbial transplantation, or metabolite-based interventions. By bridging traditional abundance-based analyses with cutting-edge computational tools, this integrated strategy refines biomarker discovery and underlines the importance of validating candidate biomarkers in independent cohorts. Harnessing the combined strengths of established biological methods and advanced machine learning offers a clearer path toward reproducible targets, promising improved early diagnosis, personalized treatments, and better outcomes for pediatric IBD patients.

As we continue to refine these computational methods, future work may delve deeper into functional characterizations of the identified taxa, examine temporal dynamics of the pediatric microbiome, and assess the efficacy of targeted interventions informed by these biomarkers. By doing so, we move closer to an era where precision medicine—fueled by robust microbial biomarkers and guided by integrative analytic frameworks—can transform the management of pediatric IBD.

## Data Availability

RAW data available at the link: https://ngdc.cncb.ac.cn/gsa-human/browse/HRA010328. According to local legal requirements and the need to protect minors, access to the raw data can be obtained through a request to the China National Information Center. Request link: https://ngdc.cncb.ac.cn/gsa-human/request/HRA010328.
